# Complete Genome Sequence of a Cytomegalovirus Towne-BAC (Bacterial Artificial Chromosome) Isolate Maintained in *Escherichia coli* for 10 Years and Then Serially Passaged in Human Fibroblasts

**DOI:** 10.1128/genomeA.00693-13

**Published:** 2013-09-26

**Authors:** Teal Brechtel, Molly Tyner, Ritesh Tandon

**Affiliations:** Department of Microbiology, University of Mississippi Medical Center, Jackson, Mississippi, USA

## Abstract

Here, we present the complete genome sequence of a cytomegalovirus, the Towne-BAC (bacterial artificial chromosome) isolate, which was maintained in bacterial cells for 10 years and then serially passaged in human fibroblasts for 10 passages. A total of 132 nucleotide differences were discovered in the Towne sequence compared to the reference sequence (GenBank accession no. AC146851).

## GENOME ANNOUNCEMENT

Several herpesviruses have been cloned in a bacterial artificial chromosome (BAC) to allow for rapid genetic engineering using the bacterial recombination system (1, 2). The virus BAC is later harvested from bacteria and subsequently transfected into eukaryotic cells for the propagation of the virus. The BAC sequence is retained within the viral genome during virus propagation; however, it can be engineered to splice out upon delivery into mammalian cells (3). The presence of a BAC sequence may lead to unwanted recombination events and instability in the virus genome (4–6). Thus, we sequenced and assembled the complete genome sequence of a cytomegalovirus Towne-BAC isolate that was maintained in *Escherichia coli* for 10 years and then serially passaged in human fibroblasts for 10 passages.

Cloning of the green fluorescent protein (GFP) and BAC sequences in the cytomegalovirus Towne strain (ATCC VR-977) genome has been reported earlier (7). We obtained the *E. coli* strain DY380 containing Towne-BAC from the laboratory of Fenyong Liu, University of California, Berkeley. This Towne-BAC isolate has been maintained in *E. coli* since the year 2003 and harvested occasionally to transfect into primary human foreskin fibroblasts (HF). The individual virus plaques in HF were purified and grown into Towne-BAC virus stocks. The genome sequence reported here belongs to a Towne-BAC virus passaged 10 times in HF. Virus DNA was purified from pelleted virions from the cleared cell culture medium using a DNeasy tissue kit (Qiagen, Inc). The quantity and quality of purified double-stranded DNA (dsDNA) were determined using a Qubit fluorometric assay (Invitrogen). The Nextera XT DNA sample preparation kit (Illumina) was used to prepare the multiplexed paired-end libraries (2 × 150 bp) before sequencing using an Illumina MiSeq. The FastQ files were imported into the CLC Genomics Workbench 6.0. The reads were trimmed for quality by using a limit of 0.05 (P of error equivalent to Q13) and a maximum number of ambiguities of 2 and discarding reads of <15 bp. The *de novo* assembly used a word size of 45, a bubble size of 90, and a minimum contig length of 1,000. Paired distances were autodetected and the reads were mapped back onto contigs. All other parameters were defaults. A total of 19.8 million paired-end reads were collected. The number of scaffolds, scaffold N_50_, and total sequence length were 4, 49 kb, and 232 kb, respectively. Each scaffold from each virus had an average coverage of >7,000×. Gaps in the genome sequence were filled by PCR and sequencing of resultant amplicons. Open reading frames were identified using an NCBI-BLAST search and were annotated in Sequin 12.30.

The 233,028-bp Towne-BAC genome revealed a total of 132 nucleotide differences in the Towne sequence compared to the reference genome (GenBank accession no. AC146851). These nucleotide differences were distributed in the RNA4.9, TRL, RL6, TRS1, and UL145 regions of the genome.

### Nucleotide sequence accession number.

This whole-genome sequence of the Towne-BAC passaged virus has been deposited at GenBank under the accession no. KF493877.
